# EBV-Induced CXCL8 Upregulation Promotes Vasculogenic Mimicry in Gastric Carcinoma *via* NF-κB Signaling

**DOI:** 10.3389/fcimb.2022.780416

**Published:** 2022-03-07

**Authors:** Jing-yue Zhang, Yu Du, Li-ping Gong, Yi-ting Shao, Jing-yun Wen, Li-ping Sun, Dan He, Jin-rui Guo, Jian-ning Chen, Chun-kui Shao

**Affiliations:** ^1^ Department of Pathology, The Third Affiliated Hospital, Sun Yat-sen University, Guangzhou, China; ^2^ Hospital of Stomatology, Guanghua School of Stomatology, Sun Yat-sen University, Guangzhou, China; ^3^ Department of Medical Oncology, The Third Affiliated Hospital, Sun Yat-sen University, Guangzhou, China

**Keywords:** RPMS1, CXCL8, NF-κB, vasculogenic mimicry, EBV-associated gastric carcinoma

## Abstract

Epstein–Barr virus (EBV)-associated gastric carcinoma (EBVaGC) is a distinct entity with a conspicuous tumor microenvironment compared with EBV-negative gastric carcinoma. However, the exact role of EBV in gastric carcinogenesis remains elusive. In the present study, we found that EBV upregulated CXCL8 expression, and CXCL8 significantly promoted vasculogenic mimicry (VM) formation of gastric carcinoma (GC) cells. In accordance with these observations, overexpression of CXCL8 increased cell proliferation and migration of AGS and BGC823 cells, while knockdown of CXCL8 with siRNA inhibited cell proliferation and migration of AGS-EBV cells. In addition, activation of NF-κB signaling was involved in VM formation induced by CXCL8, which was blocked by NF-κB inhibitors BAY 11-7082 and BMS345541. Furthermore, EBV-encoded lncRNA RPMS1 activated the NF-κB signaling cascade, which is responsible for EBV-induced VM formation. Both xenografts and clinical samples of EBVaGC exhibit VM histologically, which are correlated with CXCL8 overexpression. Finally, CXCL8 is positively correlated with overall survival in GC patients. In conclusion, EBV-upregulated CXCL8 expression promotes VM formation in GC *via* NF-κB signaling, and CXCL8 might serve as a novel anti-tumor target for EBVaGC.

## Introduction

Epstein–Barr virus (EBV) is a human oncogenic virus that infects >90% of the global population (Young et al., 2016). EBV infection is associated with a range of lymphoid and epithelial malignancies, such as Burkitt lymphoma (BL), Hodgkin lymphoma (HL), nasopharyngeal carcinoma (NPC), EBV-associated gastric carcinoma (EBVaGC), among others. Unlike NPC, which exhibits a striking geographic distribution, with high incidence rates in Southern China and Southeast Asia, EBVaGC shows no apparent geographic distribution (Young et al., 2016). About 10% of gastric carcinoma (GC) worldwide is associated with EBV (Murphy et al., 2009), and EBV plays an important role in the development of EBVaGC (Morales-Sanchez and Fuentes-Panana, 2017). EBV has been shown to change the host gene expression profiles and signaling networks to endow cancerous properties, including increased cell survival, proliferation, invasion, angiogenesis, and immune evasion (Kong et al., 2010; Dawson et al., 2012; Elgui de Oliveira et al., 2016; Lin et al., 2018). However, EBV infection does not lead to malignant transformation of normal gastric epithelial cells (Yang et al., 2020), raising uncertainty about the causal role of EBV in gastric carcinogenesis.

The tumor microenvironment is an indispensable factor in the pathophysiology of cancers, which consists of inflammatory cells, stromal cells, vasculatures, and extracellular matrix (Hinata et al., 2020). During the development of the tumor, angiogenesis plays a vital role during tumor growth and progression. It is regarded as a key step involved in tumor invasion and metastasis (Zhang et al., 2011). Besides, vasculogenic mimicry (VM) emerges as another critical vasculogenic mechanism in cancer, which plays an essential role in cancer metastasis and actively participates in cancer growth. VM is a new tumor paradigm that is independent of endothelial cells (ECs), which is identified in various malignant tumors, including ovarian (Ayala-Domínguez et al., 2019), lung (Williamson et al., 2016), gastric (Li et al., 2010), and prostate cancers (Liu et al., 2012). The channel of VM is lined by tumor cells and red blood cells appear in these channels. There are periodic acid–Schiff (PAS)-positive extracellular matrix (ECM) surrounding these channels (Delgado-Bellido et al., 2017).

Inflammatory cytokines derived from tumor cells play an important role in tumor VM formation. Studies showed that the chemokines IL-6 and IL-33 induced VM in gallbladder cancer (Pan et al., 2020) and melanoma (Yang et al., 2019), respectively. Accumulating evidence has indicated a significant role of cytokines secreted by EBV in mediating tumor growth and metastasis. In the clinical NPC samples, the expression of chemokines, such as CXCL8 (IL-8), TNF alpha, CCL-20 (MIP-3a), CCL-2 (MCP-1), and CXCL10 (IP-10), has been reported (Klein et al., 1996; Ren et al., 2004; Hsu et al., 2008; Lo et al., 2013). The overexpression of chemokines was frequently used as biomarkers, which was correlated with tumor metastasis and patient survival. Cai et al. showed that LMP1 promoted the expression of the Nod-like receptor family protein 3 (NLRP3) inflammasome, COX-2, and p-p65, further increasing the production of IL-1β, IL-6, and GM-CSF (Cai et al., 2017). These results indicated that chemokines produced by EBV-positive cells might play a role in the development of cancer. However, the roles of cytokines and their relationship with VM in EBVaGC have not yet been demonstrated.

In this study, to investigate whether EBV could promote VM formation in EBVaGC and explore the possible role of tumor microenvironment in the progression of EBVaGC, EBV-infected GC cells were established and compared with the uninfected GC cells (Marquitz et al., 2012; Ding et al., 2013). We found that CXCL8 was upregulated in EBV-infected GC cells, and overexpression of CXCL8 derived from EBV-infected cells promoted VM formation in GC cells through the NF-κB signaling, suggesting that the CXCL8 might serve as a potential therapeutic target for EBVaGC.

## Materials and Methods

### Patients and Tissue Samples

Formalin-fixed paraffin-embedded tissues from patients with EBVaGC (70 cases) and EBVnGC (62 cases) were collected between January 2010 and December 2018 at Third Affiliated Hospital of Sun Yat-Sen University. Clinical data were retrieved from patients’ medical records. Overall survival (OS) time was determined from the date of surgery to the date of death or the last follow-up visit. This study was approved by the Institutional Review Board of Third Affiliated Hospital of Sun Yat-Sen University.

### Establishment of EBV-Infected Cells

The human GC cell lines AGS and BGC823 were obtained from the Cell Bank of Type Culture Collection of Chinese Academy of Sciences (Shanghai, China). The human BL cell line Akata-EBV-GFP was provided by Prof. Mu-sheng Zeng from Cancer Center, Sun Yat-Sen University. Akata-EBV-GFP cells were modified to produce recombinant EBV, which were resistant to neomycin and could express green fluorescent protein (GFP) (Shimizu et al., 1996). EBV-infected cells (AGS-EBV and BGC823-EBV) were obtained by co-culture AGS or BGC823 with Akata-EBV-GFP using the cell-to-cell infection method as described before (Imai et al., 1998). G418 (400 μg/mL) was used to select and maintain the EBV-infected cells. The infected cells were observed for GFP expression under a fluorescence microscope (Nikon, Japan) and flow cytometry analyses. The above cells were maintained in Roswell Park Memorial Institute (RPMI) 1640 medium (Gibco, Carlsbad, CA, USA) supplemented with 10% fetal bovine serum (FBS; Gibco). All the cells were cultured at a humidified incubator with 5% CO_2_ at 37°C. All cell lines were routinely tested for mycoplasma contamination.

### RNA Extraction and qRT-PCR

Total RNA was extracted from cultured cells using TRIzol reagent (Invitrogen, Carlsbad, CA, USA) according to the manufacturer’s instructions. RNA was reverse transcribed to cDNA using RT Premix (Takara, Tokyo, Japan). Then, qRT-PCR was performed on cDNA samples using SYBR Premix Ex Taq (Takara). The relative mRNA levels of target genes were normalized to GAPDH mRNA levels, and the comparative Ct (ΔΔCt) method was used. The sequences of the specific primers used in this study are shown in **Supplementary Table S1**.

### RNA Sequencing

Total RNA was extracted from cultured cells using TRIzol reagent (Invitrogen). RNA quality was evaluated using the Agilent 2100 bioanalyzer. RNA-seq libraries were prepared using a NEBNext Ultra II Directional RNA Library Prep kit according to the manufacturer’s instructions. The reads were first mapped to the latest UCSC transcript set using Bowtie2 version 2.1.0, and the gene expression level was estimated using RSEM v1.2.15. Trimmed mean of M-values (TMM) was used to normalize the gene expression. Differentially expressed (DE) genes were identified using the edgeR program. Genes showing altered expression with P<0.05 and more than 1.5-fold changes were considered differentially expressed.

### GO Analysis

GO analysis (http://www.geneontology.org) was performed to set up gene annotations. DE transcriptome was classified into GO terms, including biological process (BP), cellular component (CC), and molecular function (MF). Fisher’s exact test was applied for the GO analysis with significant P-value calculated, and FDR was used to correct the P-values.

### KEGG Pathway Analysis

KEGG (https://www.kegg.jp) pathway analysis was adopted to describe the genes’ attributes. We turn to the Fisher’s exact test to select the significant pathway, and the threshold of significance was defined by FDR < 0.05.

### Western Blotting

Total proteins were extracted using RIPA lysis buffer (Beyotime, Haimen, China) according to the manufacturer’s protocol. Twenty micrograms of total proteins were separated on 10% SDS-PAGE gels. The proteins were transferred to PVDF membranes and then probed with primary specific antibodies overnight. Phospho-p65 (Ser536) (3033, 1:1000), p65 (8242S, 1:1000), phospho-IκBα (Ser32) (2859,1:1000), IκBα (4814, 1:1000), β-Actin (4970, 1:2000) were obtained from Cell Signaling Technologies (Beverly, MA, USA). After incubated with HRP-conjugated secondary antibodies (Beyotime) for 1 h, the signals were detected by electrogenerated chemiluminescence (ECL) detection reagents (Millipore). Relative target protein expression levels were normalized to β-Actin and visualized using ImageJ software.

### ELISA

Briefly, cells were cultured in six-well plates at a density of 5 × 10^5^ cells/well, and cell supernatants were harvested at 48 h and microfuged at 1500 rpm for 5 min to remove particles, and the supernatants were frozen at −80°C until use. The concentration of CXCL8 in the cell supernatants was determined by ELISA (R&D Systems, Abingdon, UK), using the standard curve method, according to the manufacturer’s instructions. Absorbance was measured at 450 nm using a microplate reader.

### VM Formation

A total of 40 µl Matrigel (BD Biosciences, USA) was added into a 96−well plate and cultured at 37˚C in 5% CO_2_ to polymerize. 5 × 10^4^ cells were added to the Matrigel layer and cultured with different treatments. Following 12 h of incubation for GC cells, tubules were photographed under a microscope (Nikon, Japan) and evaluated using ImageJ software. In treated groups, AGS and BGC823 cells were pretreated with BAY 11-7082 (5µM; MedChemExpress, NJ, USA) or BMS345541(5μM; MedChemExpress, NJ, USA) for 6 h.

### Cell Proliferation Assay

Cell proliferation was evaluated using the EdU kit (Beyotime) according to the manufacturer’s instructions. Briefly, 1×10^5^ cells were seeded in 12-well plates. Cells were treated with EdU reagent (10 μM) and incubated for 2 h at 37°C. The EdU+ cells were observed under a fluorescence microscope (Nikon, Japan) and evaluated using ImageJ software.

### Wound Healing Migration Assay

Wound healing migration assay was performed as previously described (Molinie and Gautreau, 2018). Briefly, cells were cultured in a six-well plate at a density of 5 ×10^5^ cells/well. After confluence, the AGS and BGC823 were scratched by a straight line using a sterile pipette tip. After that, PBS was utilized to wash cells for removing cell debris three times. The photographs of the scratch wound were recorded at 12, 24, and 36 hours to investigate and analyze the cell migration ability. An inverted microscope (Nikon, Japan) was used to obtain digital photographs. The scratch area was measured using the ImageJ software.

### IHC and PAS Staining

Immunohistochemical (IHC) staining was performed on 4 μm-thick sections of formalin-fixed paraffin-embedded tissues with an anti-CXCL8 antibody (HPA057179, 1:100; Sigma–Aldrich, MO, USA), anti-CD34 antibody (ab81289, 1:500; Abcam, Cambridge, UK), and anti-Ki67 antibody (9449S, 1:500; Cell Signaling Technology). For CD34/PAS double staining, which was used to examine VM structures, after IHC staining for CD34 described above, the sections were washed with running water for 5 min, incubated with PAS for 20 min, and counterstained with hematoxylin. All IHC slides were analyzed independently by three experienced pathologists. The CXCL8 IHC staining results were scored as follows: staining intensity score, 0 (no staining), 1 (weak), 2 (moderate), or 3 (strong); staining area score, 0 (≤10%), 1 (11–50%), 2 (51–75%), and 3 (≥75%). The staining intensity score and staining area score were then multiplied to yield a final score. Finally, it was divided into two groups: low expression (final score ≤ 3) and high expression (final score > 3).

### siRNAs

siRNAs were synthesized by Guangzhou RiboBio Co., Ltd., and the sequences were as follows: siRNA targeting RPMS1#1: 5′-GGCAAGGUCCGGCGUGUCCACdTdT-3′; siRNA targeting RPMS1#2: 5′- UCGUCGACGGGCAAGGUCCGGdTdT-3′; siRNA targeting RPMS1#3: 5′-GACGGGCAAGGUCCGGCGUGUdTdT-3′. siRNA targeting CXCL8#1: 5′-CTTAGATGTCAGTGCATAA-3′; siRNA targeting CXCL8#2: 5′-GTCAGTGCATAAAGACATA-3′. Lipofectamine RNAiMAX (Invitrogen) was used for siRNA transfection according to the manufacturer’s instructions.

### *In Situ* Hybridization

*In situ* hybridization (ISH) assay was performed with a commercially available EBV oligonucleotide probe complementary to EBER-1 (PanPath, Amsterdam, Netherlands), as previously described by Chen et al. (2010). Sections from a known EBER-1-positive NPC tissue were used as the positive controls and a sense probe for EBER-1 was used as the negative control.

### Animal Studies

Female BALB/c nude mice (4–6 weeks old) were obtained from GemPharmatech Laboratory (Nanjing, China). For the xenograft model, 5 × 10^6^ cells were injected into the subcutaneous tissue of the mouse (n = 5 per group). All mice were sacrificed after four weeks, and the xenografts were fixed with phosphate-buffered formalin and sectioned for H&E staining and immunohistochemical analysis. All animal studies were performed in accordance with the institutional ethics guidelines for the animal experiments, which were approved by the Experimental Animal Ethics Committee of the Third Affiliated Hospital, Sun Yat-sen University.

### Statistical Analysis

Statistical analyses were performed using SPSS (version 22.0; IBM Corp., Armonk, NY, USA). Spearman’s correlation test or unpaired Student’s *t* test was used when appropriate. Survival analyses were performed using the Kaplan-Meier method and a log-rank test. Data with a non-Gaussian distribution were compared with a two-tailed Mann–Whitney test. A *p* value less than 0.05 was considered statistically significant.

## Results

### Upregulation of CXCL8 in EBV-Positive GC Cells

**Figure 1:**
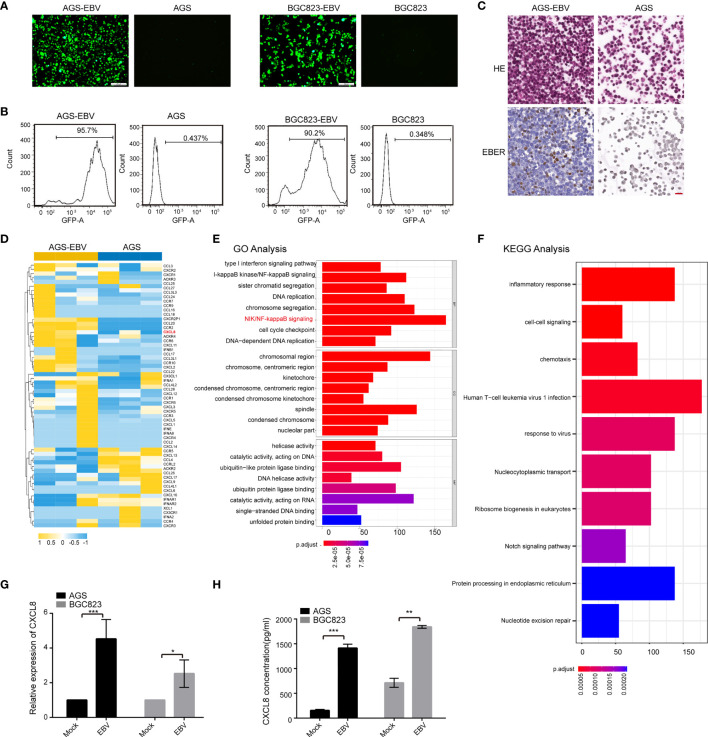
CXCL8 was upregulated in EBV-positive gastric carcinoma cells. **(A)** Established EBV-infected GC cell lines. Co-culture of AGS or BGC823 and EBV-positive Akata cells was adopted to construct EBV-positive cells (see Methods). After G418(400μg/ml) selection, EBV-positive cells were obtained. Scale bars=100 µm. **(B)** Upon infection of EBV into GC cells containing GFP virions, an ~90% of GFP fluorescence was observed *via* flow cytometry. **(C)** EBER *in situ* hybridization assay confirmed the presence of EBV in the majority of EBV-infected cells. **(D)** Heatmap depicting FPKM values for cytokines in AGS and AGS-EBV cells. Of note, CXCL8 was upregulated in AGS-EBV compared to AGS. **(E)** The top significant enrichment pathways according to GO analysis of the differential expressed cytokines between AGS and AGS-EBV cells are shown. The differential expressed cytokines were mainly related to anti-virus immunity and NF-κB signaling pathway. **(F)** The KEGG pathway analysis showed the differential expressed cytokines between AGS and AGS-EBV cells were related to the biological processes of immune response and chemotaxis. **(G)** Relative mRNA expression of CXCL8 was assessed in paired EBV-negative and EBV-positive GC cells (AGS vs. AGS-EBV and BGC823 vs. BGC823-EBV) by qRT−PCR. CXCL8 mRNA level was higher in EBV positive cells than that in EBV negative cells. **(H)** CXCL8 concentration in the supernatant of EBV− and EBV+ GC cells was quantified by ELISA at indicated times. CXCL8 level was higher in EBV positive cells than that in EBV negative cells. *p<0.05, **p<0.01, ***p<0.001.

To investigate the role of EBV in gastric carcinogenesis, we first established EBV-infected GC cell lines as previously described (Yue et al., 2019). Two GC cell lines, AGS and BGC823, were infected with recombinant EBV derived from the BL cell line Akata. Upon G418 selection (400 μg/ml), the GFP fluorescence was observed via fluorescence microscopy and flow cytometry analyses (**Figures 1A, B**). EBER in situ hybridization confirmed the presence of EBV in the majority of cells in the EBV-infected cells (**Figure 1C**). To gain mechanistic insights into EBV carcinogenesis, RNA-seq analysis was performed in AGS and AGS-EBV cells. Here, we focused on cytokines expression, so we compared the expression profiles of the 92 known cytokines between AGS and AGS-EBV cells. In total, 21 DE cytokines (fold change ≥ 2) were identified and CXCL8 displayed the high expression in AGS-EBV group (**Figure 1D**). GO enrichment analysis of the DE cytokines between AGS and AGS-EBV cells showed that these cytokines involved in processes including NF-kappaB (NF-κB) signaling and type I interferon signaling pathways (**Figure 1E**). KEGG analysis showed that the DE cytokines were enriched in biological processes of immune response and chemotaxis (**Figure 1F**). These findings indicated that the change of cytokines expression in AGS-EBV might contribute to anti-virus immunity, and NF-κB signaling pathway might drive EBV carcinogenesis.

The expressions of CXCL8 in the paired EBV-positive and EBV-negative cells (AGS-EBV vs. AGS and BGC823-EBV vs. BGC823) were verified by qRT−PCR analysis. CXCL8 mRNA expression was significantly higher in EBV-positive cells than in EBV-negative cells (**Figure 1G** and **Figure S1**). Quantitative ELISA showed that CXCL8 concentration in the supernatant of EBV-infection cells was higher than that in parental cells (**Figure 1H**). Western blotting results showed that the expression of CXCL8 is higher in EBV-infective cells than that in parental cells (**Figure S2**). Moreover, we performed qRT−PCR and ELISA assays using EBV-positive GC cell lines (SNU719 and YCCEL1 cells), the results showed that the level of CXCL8 was significantly upregulated in SNU719 and YCCEL1 cells compared to AGS and BGC823 cells (**Figure S3**). Taken together, these data showed that EBV infection led to the upregulation of CXCL8 in GC cells.

### EBV-Upregulated CXCL8 Expression Promotes VM Formation *In Vitro*

**Figure 2:**
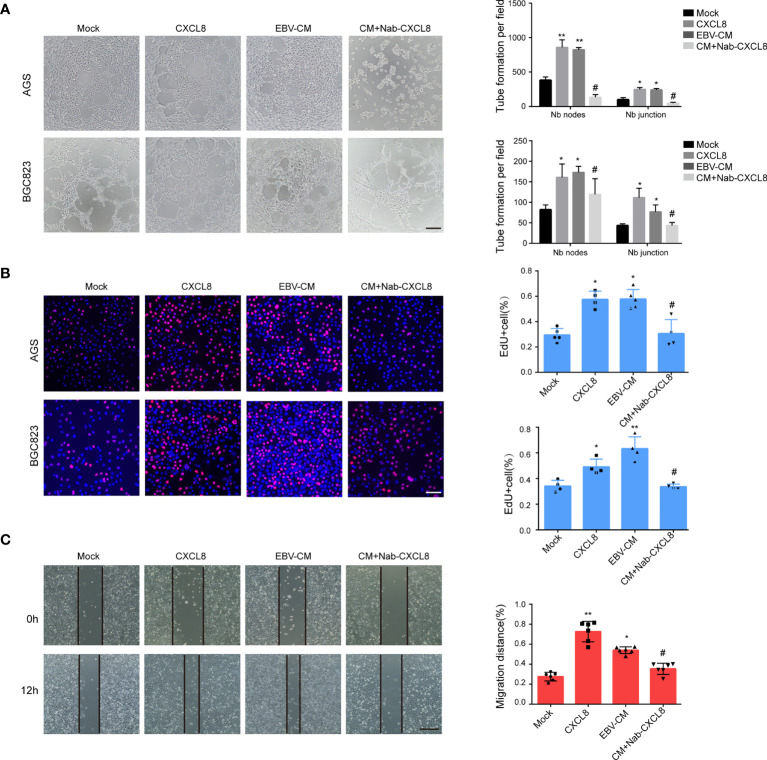
CXCL8 promoted vasculogenic mimicry formation *in vitro*. **(A)** Images (left) and quantification (right) of tube formation in cells with different treatments as indicated. Images were taken 12 h after seeding on Matrigel. Tubes were counted with 100× magnification by Image J. CXCL8:2ng/ml; EBV-CM: EBV conditioned medium; Nab-CXCL8: anti-human CXCL8 neutralization antibody (Nab),1 μg/mL for neutralization; Nb: Number branches. CXCL8 and EBV-CM promoted VM formation in EBV-positive cells, blocking CXCL8 by neutralization antibody (Nab) decrease the tube formation in GC cells. Scale bars = 50 μm. **(B)** EdU assay assessed the proliferation of AGS and BGC823 cells with different treatments as indicated. Scale bars = 50 μm. CXCL8 promoted cells proliferation in EBV-positive cells, so as EBV-CM, which was reversed by CXCL8 neutralization. **(C)** Migration of AGS and BGC823 cells with different treatments as indicated were measured by wound healing assays. CXCL8 promoted cells migration in EBV-positive cells, so as EBV-CM, which was reversed by CXCL8 neutralization. Scale bars=100 µm. Compared with NC: *P<0.05, **P<0.01; Compared with CXCL8 or EBV-CM: ^#^P<0.01.

In addition to the well-known chemotaxis effects on immune cells, CXCL8 has exhibited its promoting effects on angiogenesis in pancreatic cancer (Matsuo et al., 2009; Pausch et al., 2020) and VM formation in glioblastoma (Angara et al., 2018). Therefore, the association between CXCL8 and VM formation in GC was evaluated. As shown in **Figure 2A** and **Figure S4**, the addition of recombinant human CXCL8 (2 ng/ml, 12 h) promoted VM formation in GC cells, similar to the addition of conditioned medium of AGS-EBV (EBV-CM). The decrease of CXCL8 by neutralization CXCL8 antibody (Nab-CXCL8, 1 μg/ml) in GC cells significantly decreased the number of tubes formed on Matrigel. Furthermore, we performed PAS staining to observe the tubular structures between EBV-negative and EBV-positive cells. PAS staining results showed that EBV-positive cells exhibited more VM structures than EBV-negative cells (**Figure S5**).VM formation is associated with cell proliferation and migration; thus, the effects of CXCL8 on cell proliferation and migration in GC cells was further assessed. The proliferation of AGS and BGC823 cells was increased both in the CXCL8 and EBV-CM groups, but decreased in the anti-CXCL8 group (**Figure 2B**). Wound healing migration assays revealed that the addition of recombinant human CXCL8 or EBV-CM increased the cell migration ability of AGS and BGC823 cells. The effects were reversed by being treated with anti-CXCL8 (**Figure 2C** and **Figure S6**). Furthermore, knockdown of CXCL8 with siRNA blocked VM formation as well as cell proliferation and migration (**Figure S7**). These results indicated that EBV-upregulated CXCL8 promoted VM formation of GC cells.

### CXCL8 Promotes VM Formation *Via* the NF-κB Signaling

**Figure 3:**
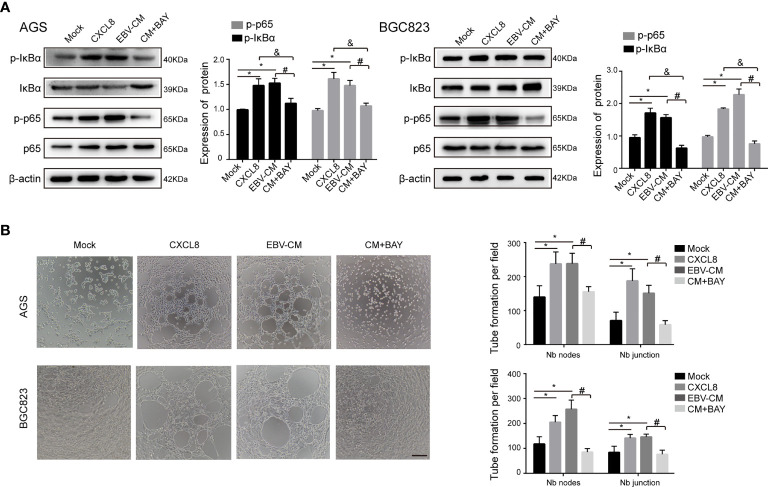
NF-κB signaling participated in VM formation induced by CXCL8. **(A)** The expressions of NF-κB signaling molecules in GC cells with different treatments as indicated were determined by immunoblotting. The expression of p−IκBα and p−NF-κB in AGS and BGC823 cells was upregulated with treatment of recombinant human CXCL8 as well as EBV-CM, which was reversed by BAY (5 µM). BAY: BAY11-7082, a NF-κB inhibitor. β-Actin was used as a loading control. **(B)** Representative images (left) and quantification (right) of tube formation on Matrigel with different treatments as indicated. BAY 11-7082 (5 µM) decreased VM formation. Nb: Number branches. Tube numbers were counted with 100× magnification by Image J Scale bars = 50 μm. Compared with NC: *P<0.05; compared with +CXCL8 and +EBV-CM: ^#^P<0.01; compared with BAY11-7082: ^&^P<0.01.

CXCL8 has been revealed to bind to its receptor and then trigger many downstream signaling cascades, among which NF-κB signaling is associated with tumor VM formation (Zhao et al., 2017). As aforementioned, we demonstrated that the DE cytokines between AGS and AGS-EBV cells were enriched in NF-κB signaling pathway (**Figure 1E**). Therefore, we detected the molecules in the NF-κB singling pathway by Western blotting. As shown in **Figure 3A**, the expressions of p-IκBα and p-p65 in AGS and BGC823 cells were upregulated with the treatment of recombinant human CXCL8 as well as EBV-CM, whereas the expressions of total IκBα and p65 in AGS and BGC823 cells remained unchanged. When treated with BAY 11-7082(5 μM), an inhibitor of NF-κB, the expression of p−p65 and p-IκBα were downregulated in CXCL8-treated AGS and BGC823 cells (**Figure 3A**). In accordance with these observations, both recombinant human CXCL8 and EBV-CM increased the number of Matrigel VM formation of EBV-positive cells, which was reversed by inhibition of NF-κB signaling (**Figure 3B**). Besides, we investigated the effect of the NF-kB inhibitor BAY 11-7082 on CXCL8 production in YCCEL1, AGS-EBV, and BGC823-EBV cells, and found that the CXCL8 showed a significant reduction in BAY 11-7082 group (**Figure S8**). We also performed another NF-kB inhibitor (BMS345541) to support the involvement of the NF-kB pathway in the capacity of CXCL8 to regulate VM formation, and found that the expression of p-IKKα/β, p−p65, and p−IκBα was inhibited with the treatment of inhibitor BMS345541 (**Figure S9A**). In addition, the number of tubules in the EBV-CM group was significantly increased compared with the control groups and significantly decreased when treated with BMS345541 (**Figure S9B**). This indicated that NF-κB signaling participated in VM formation induced by CXCL8 in GC. In addition to NF-κB signaling, previous studies also reported that ERK phosphorylation and STAT3 phosphorylation might be involved in the CXCL8 expression (Huang et al., 2017; Bae et al., 2019). Therefore, we also detected the ERK phosphorylation and STAT3 phosphorylation levels in EBV-infected GC cells and their parental cells. The results showed that both levels in EBV-positive GC cells were similar to the EBV-negative control cells (**Figure S10**). Additionally, EBV-infection did not affect VEGF expression, indicating that VEGF did not contribute to the EBV-induced VM. These findings indicated that NF-κB signaling participated in VM formation induced by CXCL8 in GC.

To further confirm the promoting effects of CXCL8 on VM formation and explore the underlying mechanisms, we investigated whether CXCL8 could regulate the expressions of VM-promoting genes MMP1, MMP9 and Twist. We found that MMP1, MMP9, and Twist expressions were higher in AGS cells treated with recombinant human CXCL8 or EBV-CM (**Figure S11**).

### RPMS1 Is Involved in VM Formation and CXCL8 Upregulation

**Figure 4:**
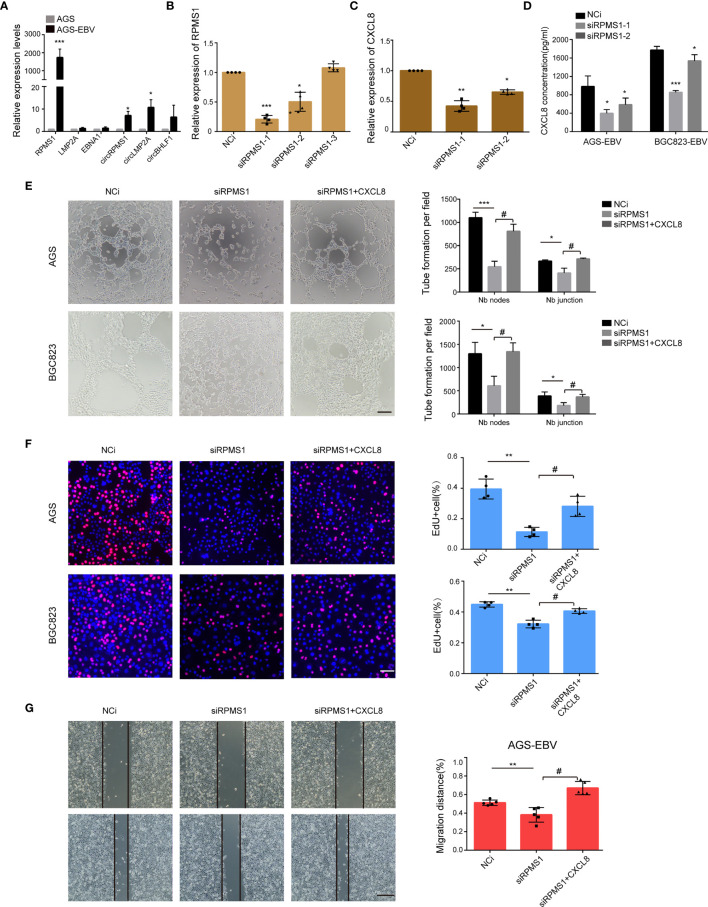
RPMS1 was involved in VM formation. **(A)** qRT-PCR analysis of EBV latent genes in AGS and AGS-EBV cells carrying recombinant EBV. RPMS1 mRNA level was higher in EBV positive cells than that in EBV negative cells. GAPDH was used as the internal control. **(B)** AGS-EBV cells were transfected with control or RPMS1-specific siRNAs. The knockdown efficiency was determined by qRT-PCR. Among the three siRNAs, si-1 and si-2 dramatically downregulated RPMS1 expression. **(C, D)** CXCL8 level were determined by qRT-PCR and ELISA. CXCL8 downregulation in the RPMS1 knockdown groups. **(E)** AGS-EBV and BGC823-EBV cells were transfected with NC or siRNA RPMS1. Representative images (top) and quantification (bottom) of tube formation on Matrigel of control and RPMS1 knockdown EBV positive cells. Tubes were counted with 100× magnification by Image J Scale bars = 50 μm. **(F)** EdU assay assessed the proliferation of AGS-EBV cells transfected with control or RPMS1-specific siRNA. Scale bars = 50 μm. **(G)** Migration of AGC-EBV cells transfected with control or RPMS1-specific siRNA were measured by wound healing assays. Scale bars=100 µm. Compared with NC: *P<0.05, **P<0.01, ***p<0.001; compared with siRPMS1: ^#^P<0.01.

Having noted the role of EBV-induced CXCL8 in promoting VM, we next tried to identify the viral genes that contribute to VM formation. We first examined the EBV-encoded gene expression in EBV-infected GC cell lines. qRT-PCR results suggested that LMP1, EBNA3C, BHLF1, and BZLF1 were not expressed in AGS-EBV cells, and RPMS1, EBNA1, LMP2A, ebv-circRPMS1, ebv-circLMP2A, and ebv-circBHLF1 were all expressed in AGS-EBV cells, among which RPMS1 transcript was the highest (**Figure 4A**). Among these latent genes, RPMS1, an EBV BART Long Non-coding RNAs, have been implicated in cytokine expression (Verhoeven et al., 2019). Upon transfection of RPMS1 siRNA into AGS-EBV cells, qRT-PCR showed a significant reduction of RPMS1 and CXCL8, but no effect on other EBV latent genes (circRPMS1, EBNA1, and LMP2A) (**Figures 4B, C** and **Figure S12**). ELISA also indicated CXCL8 decreased in EBV-positive GC cells following RPMS1-knockdown (**Figure 4D**). More importantly, knockdown of RPMS1 with siRNAs reduced the tube-forming capacities of EBV-infected GC cells, which could be reversed by being treated with CXCL8(2 ng/ml) (**Figure 4E**). Furthermore, RPMS1 knockdown in EBV-positive cells inhibited cell proliferation and migration, which was also reversed by treatment with CXCL8 (**Figures 4F, G**, and **Figure S13**). These results suggested an important role of RPMS1 in EBV-induced VM formation.

Then, we investigated how RPMS1 regulates CXCL8 expression. ChIP analysis with anti-H3K27me showed a higher enrichment at the promoter region (−49) of the CXCL8 gene, but this higher enrichment was not observed at the end of the CXCL8 gene (+2918) (**Figure S14**). Besides, knockdown of RPMS1 resulted in significantly less H3k27me expression (**Figure S15**). These findings suggested that RPMS1 could downregulate CXCL8 expression by interfering with H3K27me-mediated transcription.

### CXCL8 Is Positively Correlated With VM *In Vivo*

**Figure 5:**
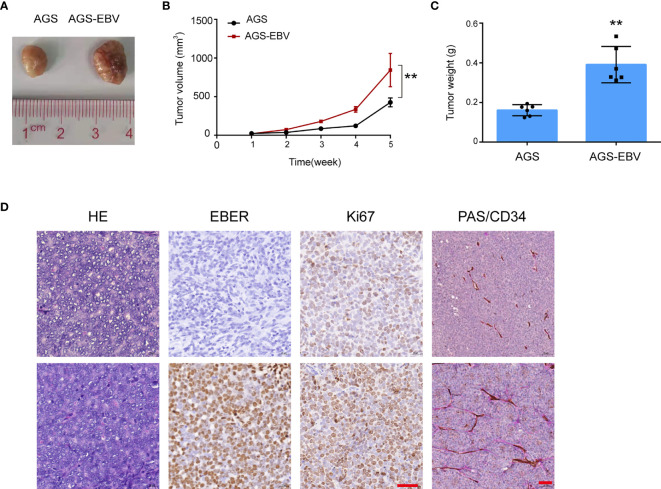
CXCL8 promoted vasculogenic mimicry formation *in vivo*. **(A)** Image of AGS and AGS-EBV xenograft tumors from BALB/c nude mice. Tumor volume in the AGS-EBV group was larger than in the AGS group, suggesting EBV promote tumor growth. **(B)** Growth curves of AGS and AGS-EBV xenograft tumors. Tumor growth in the AGS-EBV group was faster than in the AGS group, n = 6, **p< 0.01. **(C)** 28 days after injection, the tumor weights were measured. Tumor weights in the AGS-EBV group was higher than in the AGS group, n = 6. **p<0.01. **(D)** H&E, EBER, Ki67, PAS and mouse CD34 staining of AGS-EBV xenograft sections. Ki67 protein expression including the number and staining intensity of positive cells in the AGS-EBV group were significantly higher than in the AGS groups. CD34 and PAS double-Staining for VM channel, PAS-positive substances lined these channels and formed basement membrane-like structure. Scale bars = 100 μm.

To evaluate the VM formation *in vivo*, EBV− and EBV+ AGS cells were injected subcutaneously into nude mice. The xenografts images were taken four weeks post-implantation (**Figure 5A**). Compared with the parental cells, AGS cells infected with EBV dramatically exacerbated tumor growth (**Figures 5B, C**). Double staining CD34/PAS showed that the tumors derived from EBV+ cells exhibited more VM structures than those derived from EBV- cells (**Figure 5D**), suggesting that EBV infection promoted the VM formation and tumorigenicity of GC cells.

**Figure 6:**
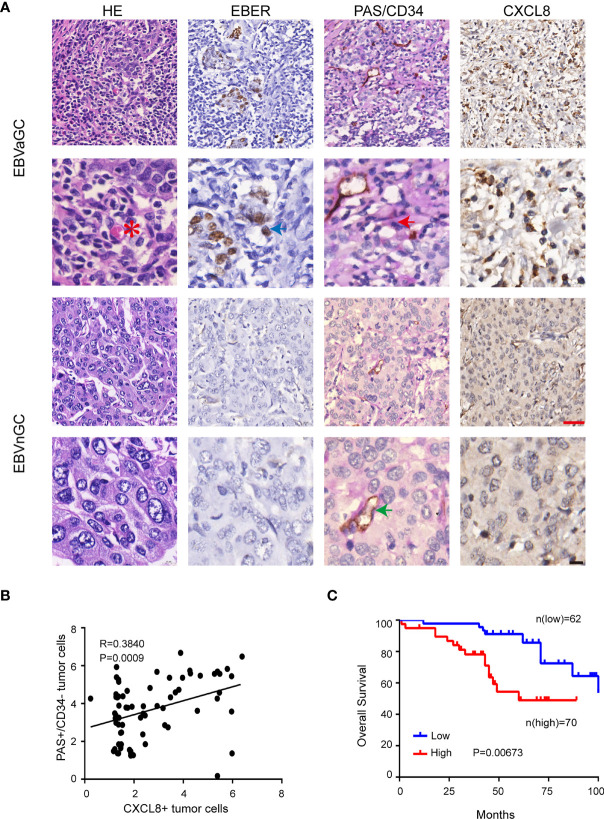
CXCL8 was positively correlated with VM in EBVaGC. **(A)** EBV-negative and EBV-positive gastric carcinoma serial sections were stained with H&E, EBER, PAS, and anti-human CD34, CXCL8, VM channel (red arrow) was formed by tumor cells and there were red cells (red star mark) in the center of the channels. Endothelial cells were stained as brown by immunohistochemical staining for CD34(green arrow), Red scale bars = 100 μm, black scale bars = 10 μm. **(B)** Pearson correlation of PAS+/CD34− tumor cells of VM with CXCL8+tumor cells in EBVaGC tissue. p<0.001, r = regression coefficient, Pearson’s correlation coefficient analysis. **(C)** Kaplan-Meier curves of overall survival according to CXCL8 expression in human gastric carcinoma samples. n = 132, P = 0.00673, log-rank test.

We also investigated the correlation between VM and CXCL8 formation in GC tissues. The VM channels positive for PAS and negative for CD34 were lined with EBER-positive tumor cells and contained red blood cells (**Figure 6A**, top panel). CXCL8 was mainly located in the nucleus of tumor cells. However, cytoplasm and membranous positivity could also be observed. As shown in **Figure 6A**, EBVaGC showed stronger CXCL8 immunostaining than EBVnGC. In addition, the PAS+CD34− GC tumor cells expressed high levels of CXCL8 in EBVaGC (**Figure 6B**). Furthermore, high CXCL8 expression was associated with worse OS in GC patients (**Figure 6C**). These data confirmed the existence of VM in GC clinical samples and further demonstrated the association between EBV infection, VM formation, and CXCL8 expression.

## Discussion

**Figure 7:**
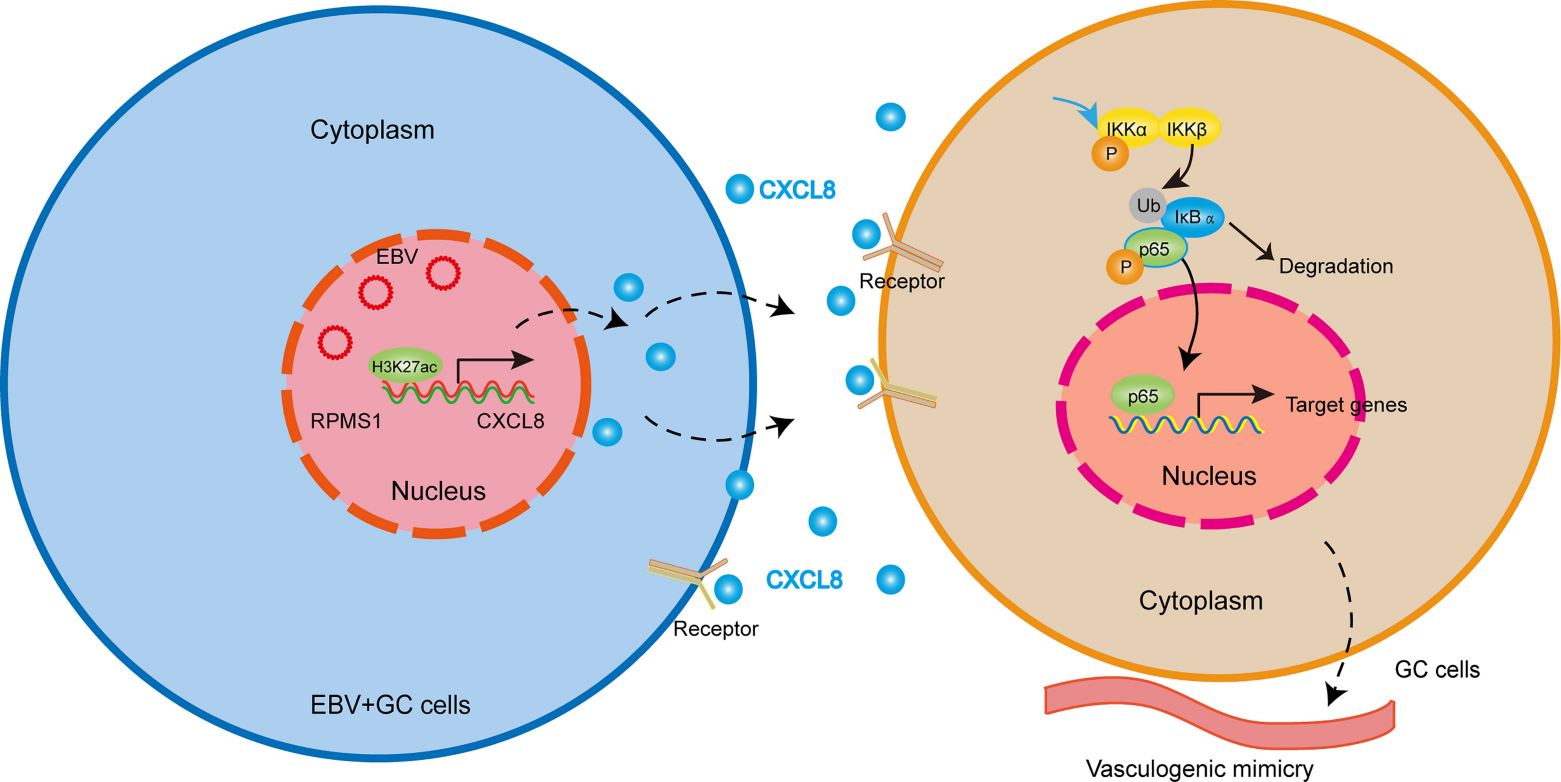
Working model illustrating the mechanism by which EBV-derived CXCL8 promotes VM and the aggressiveness of EBVaGC. EBV-encoded lncRNA RPMS1 upregulates CXCL8 expression. CXCL8 binds to its receptor, CXCR1/2, and then triggers NF-κB signaling cascade, which accelerated VM formation.

In this study, we showed that EBV-encoded lncRNA RPMS1 upregulated CXCL8 expression, which accelerated VM formation through promoting EBV-positive cells proliferation and migration. The promoting effect of CXCL8 on VM formation was reversed by the inhibitors of NF-κB signaling. Additionally, CXCL8 expression was positively correlated with VM formation and poor OS in GC patients (**Figure 7**).

It is generally believed that solid tumors require adequate blood supply with adequate oxygen for tumor growth, and VM is one of the new functional microcirculation models that can provide sufficient blood supply and plays a critical role in the spread of cancer (Maniotis et al., 1999; Zhang et al., 2011). In recent years, VM has been reported in a variety of malignant tumors, such as osteosarcoma, hepatocellular carcinoma, breast cancer, lung cancer, and GC (Luo et al., 2020). In this study, we revealed that EBV was positively correlated with VM formation in EBVaGC. Xiang et al. reported the role of EBV in promoting VM formation in NPC and GC through the PI3K/AKT/mTOR/HIF-1α axis (Xiang et al., 2018). However, their study did not explore the role of the tumor microenvironment in the regulation of EBV-induced VM formation. In the present study, we found that EBV infection led to the upregulation of CXCL8 in GC cells and CXCL8 expression promotes VM formation, which, however, was inhibited by CXCL8 knockdown, indicating that CXCL8 is involved in VM formation in EBVaGC. As a cytokine, CXCL8 is mainly responsible for the recruitment of neutrophils, basophils, and T cells. Accumulated evidence has revealed that CXCL8 promotes metastasis in many tumors, such as thyroid cancer (Song et al., 2019), prostate cancer (Baci et al., 2019), GC (Bae et al., 2019), and breast cancer (Aikins et al., 2017). Here, we showed that EBV-induced CXCL8 upregulation promotes VM formation in GC, indicating that GC cells infected with EBV could phenotypically mimic endothelial-like cells. Angara K et al. found that CXCL8 exhibited its promoting effects on angiogenesis in pancreatic cancer and VM formation in glioblastoma (Angara et al., 2018). Bae et al. reported that PTPRD-inactivation-induced CXCL8 expression promotes angiogenesis and metastasis in GC (Bae et al., 2019).

In addition, we explored the molecular mechanisms underlying CXCL8 upregulation in EBVaGC. GO enrichment analysis of the DE cytokines between AGS and AGS-EBV cells suggested that NF-κB signaling might be involved. We then verified *in vitro* that NF-κB signaling participated in VM formation induced by CXCL8, which could be reversed by NF-κB signaling inhibitors BAY 11-7082 and BMS345541. NF-κB signaling is known to be involved in VM formation, which could promote tumor development (Lo et al., 2013; Tang et al., 2017; Liubomirski et al., 2019). For example, Zhang et al. found that miR-3928v contributes to hepatocellular carcinoma malignancy by increasing VM *via* NF-κB/EGR1 pathway (Zhang et al., 2018). The activation of NF-κB signaling caused IκB degradation and p65 phosphorylation, the latter translocated to the nucleus and then bound with a consensus sequence of various genes to activate their transcription. In addition to NF-κB signaling, previous studies reported that ERK phosphorylation and STAT3 phosphorylation might also be involved in the CXCL8 expression. For instance, Huang et al. showed that c-Src and ERK were involved in the STAT3/CXCL8 induction in primary human nucleus pulposus culture (Huang et al., 2017); Bae et al. found that the increase in CXCL8 expression was mediated by both ERK and STAT3 signaling (Bae et al., 2019). Therefore, in the present study, we also evaluated the ERK phosphorylation and STAT3 phosphorylation levels in EBV-infected GC cells and their parental cells. The results showed that there was no significant difference on both levels.

It is known that MMP1, MMP9, and Twist participate in tumor invasion, metastasis, and VM formation. In this study, we found that the expression of MMP1, MMP9, and Twist1 was highly expressed in EBV-positive GC cells. In melanoma tumor cells, activation of PI3K enhanced VM formation by affecting MMP-2 and MT1-MMP activity (Hess et al., 2003). In pancreatic cancer cells, arginine starvation decreased metastasis by inhibition of MMP-1/9 (Wang et al., 2020). Interfering Twist1 inhibited epithelial-mesenchymal transition (EMT) and limited lipopolysaccharide-induced inflammation, migration, and invasion in A2058 melanoma cells with the decrease of MMP1/9, VEGF, TNF-α, and IL-6 (Ding et al., 2019). In addition, other genes have been reported to facilitate VM formation. For instance, repression of MMP2 and MMP9 could block VM formation in pancreatic ductaladenocarcinoma (Zhuo et al., 2019). PPI impaired VM formation by blocking the PI3k-Akt-Twist1-VE-cadherin pathway hepatocellular carcinoma (Xiao et al., 2018).

Although the role of EBV-induced CXCL8 in promoting VM was confirmed, which viral products contribute to VM formation? We first examined the EBV-encoded gene expression in EBV-infected GC cell lines. The results showed that LMP1, EBNA3C, BHLF1, and BZLF1 were not expressed in AGS-EBV cells, whereas RPMS1, EBNA1, LMP2A, ebv-circRPMS1, ebv-circLMP2A, and ebv-circBHLF1 were all expressed in AGS-EBV cells. In the host, EBV is known to exist in a latent state expressing only a limited set of viral gene products (known as latent products), which include EBV nuclear antigens (EBNAs 1, 2, 3A, 3B, 3C, and -LP), latent membrane proteins (LMPs 1, 2A, and 2B), EBV-encoded small non-coding RNAs (EBERs 1 and 2) and BamHI A right transcript (BARTs) (Thompson and Kurzrock, 2004; Fukayama and Ushiku, 2011). Three latency patterns, latency І/II/III, have been classified based on latent products (Fukayama and Ushiku, 2011). EBVaGC is a latency I neoplasm and expresses EBNA1, EBER, BART, and LMP2A (nearly half of EBVaGC cases) (Imai et al., 1994; Fukayama and Ushiku, 2011). Recently, studies found that some virus-derived circular RNAs are also expressed in EBVaGC (Huang et al., 2019; Gong et al., 2020). Among the latent products expressed in EBVaGC, RPMS1, an EBV BART long non-coding RNAs, have been implicated in cytokine expression (Verhoeven et al., 2019). Further *in vitro* and *in vivo* functional experiments demonstrated that RPMS1 participated in VM formation in EBVaGC. Ang Li et al. showed that RPMS1 mRNA was highly expressed in primary NPC than non-carcinoma tissue of nasopharynx and peripheral blood lymphocytes of NPC patients (Li et al., 2005). RPMS1 also negatively regulated EBNA2 and Notch activity through interactions with the CBF1-associated corepressor complex (Zhang et al., 2001). These data implied that RPMS1 played an important role in the development of EBV-associated neoplasms. Additionally, our findings that EBV was present in EBVaGC and EBV-encoded RPMS1 could induce VM formation, providing crucial evidence to clarify that EBV is an important pathogen of EBVaGC.

Most importantly, our findings also shed light on the clinical relevance of CXCL8 in GC patients. In the present study, we found that high levels of CXCL8 in GC tumors correlated with advanced tumor progression and poor patient survival. Pawluczuk et al. also reported that aberrant CXCL8 expression in gastric carcinoma was associated with poor prognosis (Pawluczuk et al., 2021). In addition, overexpression of CXCL8 enhanced growth and metastasis in human melanoma (Wu et al., 2012). Lee YS et al. revealed that CXCL8 in the tumor microenvironment stimulated cell growth, progression, and metastasis of colon cancer (Lee et al., 2012). EBVaGC cells expressed higher levels of CXCL8 relative to EBV-negative GC (EBVnGC) cells. However, it is well established that patients with EBVaGC have better prognosis relative to the ones with EBVnGC (van Beek et al., 2004). The potential reasons for these inconsistent results might be associated with EBV infection, the high CXCL8 expression in EBVaGC cells might represent the host defense against EBV infection. EBVaGC has specific histological features which show irregularly anastomosing tubules accompanied with lots of lymphocytic infiltration and result in a “lace-like” pattern at low magnification, thus plenty of lymphocytes accompanied with rich cytokines infiltration in the tumor microenvironment are the most remarkable features (Yang et al., 2020). The infiltrating CD8^+^ T cells in EBVaGC were predominant and with high cytotoxicity, it is thought to be associated with the generally favorable prognosis and lower frequency of lymph node metastases of EBVaGC (van Beek et al., 2006). In another cohort conducted by our group, we found that EBVaGC, which also showed massive lymphocytes infiltration, had a significantly better 5-year overall survival than did EBVnGC (80.0% vs. 44.9%) (Dong et al., 2016). Because the number of CXCL8 in gastric carcinomas may be influenced by some clinicopathological parameters (such as histology, TNM staging), to minimize the selection bias, 70 EBVaGC cases and 62 EBVnGC cases with matched clinicopathological parameters were selected for further investigation of CXCL8 and prognosis in the present study.

However, our study has several limitations. Firstly, we did not investigate VM formation induced by CXCL8 using an *in vivo* model, mainly because of the lack of an orthotopic EBVaGC model. Secondly, we did not investigate the direct mechanism of how RPMS1 upregulated CXCL8. Finally, we did not investigate the mechanism of p65 on VM formation as the downstream regulator of p65 is currently unknown.

In summary, we found that CXCL8 secreted by EBV-positive cells promoted the VM formation in EBVaGC *via* the NF-κB signaling pathway. These findings indicated that blocking this pathway may serve as a potential therapeutic target for EBVaGC treatment.

## Data Availability Statement

The datasets presented in this study can be found in online repositories. The names of the repository/repositories and accession number(s) can be found below: https://www.ncbi.nlm.nih.gov/geo/query/acc.cgi?acc=GSE185627.

## Ethics Statement

The studies involving human participants were reviewed and approved by the Institutional Review Board of Third Affiliated Hospital of Sun Yat-Sen University. The patients/participants provided their written informed consent to participate in this study. The animal study was reviewed and approved by the Experimental Animal Ethics Committee of the Third Affiliated Hospital, Sun Yat-sen University.

## Author Contributions

J-YZ designed the study, performed the experiments and wrote the manuscript. J-NC performed part of the experiments and revised the manuscript. YD and L-PG interpreted part of the data and revised the manuscript. DH, J-NC, and J-RG performed IHC Scoring and provided statistics of data. Y-TS and J-YW involved in data curation and formal analysis. L-PS involved in collection of clinical specimens. C-KS designed the study and revised the manuscript. All authors contributed to the article and approved the submitted version.

## Funding

This work was supported by the National Natural Science Foundation of China (82073397), the Guangdong Basic and Applied Basic Research Foundation (2019A1515011455), the Natural Science Foundation of Guangdong Province (2018A030313650), the Guangzhou Science and Technology Project (202102010156) and the NSFC cultivating grant of The Third Affiliated Hospital, Sun Yat-sen University (2020GZRPYMS01), Guangdong Province, China.

## Conflict of Interest

The authors declare that the research was conducted in the absence of any commercial or financial relationships that could be construed as a potential conflict of interest.

## Publisher’s Note

All claims expressed in this article are solely those of the authors and do not necessarily represent those of their affiliated organizations, or those of the publisher, the editors and the reviewers. Any product that may be evaluated in this article, or claim that may be made by its manufacturer, is not guaranteed or endorsed by the publisher.
