# Supplementary material for: EBV-Induced CXCL8 Upregulation Promotes Vasculogenic Mimicry in Gastric Carcinoma via NF-κB Signaling
Source: Front Cell Infect Microbiol. 2022 Mar 7;12:780416. doi: 10.3389/fcimb.2022.780416 (PMC8936189; doi:10.3389/fcimb.2022.780416)
Supplement: Supplementary file 1 [file DataSheet_1.docx]

**Table S1. Primer sequences** **used in this study**

| Gene | Forward (5'‑3') | Reverse (5'‑3') |
| --- | --- | --- |
| CXCL8 | TCTGCAGCTCTGTGTGAAGGT | TGTGTTGGCGCAGTGTGGT |
| IFNAR1 | CCTCCTGTGAGCCTAAGTGC | AAGGGCCTACCCTCAGTGTT |
| IFNAR2 | CTCCCACAGTTTCCCTGTGT | CTGGGAGCTTCTCAGGATTG |
| CXCL2 | CTCAAGAATGGGCAGAAAGC | AAACACATTAGGCGCAATCC |
| CXCL3 | GCAGGGAATTCACCTCAAGA | GGTGCTCCCCTTGTTCAGTA |
| IFNB1 | CATTACCTGAAGGCCAAGGA | CAGCATCTGCTGGTTGAAGA |
| Twist1 | GTCCGCAGTCTTACGAGGAG | CCAGCTTGAGGGTCTGAATC |
| MMP1 | GGTCTCTGAGGGTCAAGCAG | AGTTCATGAGCTGCAACACG |
| MMP9 | TTGACAGCGACAAGAAGTGG | GCCATTCACGTCGTCCTTAT |
| CXCL8-49 | GGGCCATCAGTTGCAAATC | TTCCTTCCGGTGGTTTCTTC |
| CXCL8+2918 | CCAGCTGTGTTGGTAGTGCT | AAACAAGTTTCAACCAGCAAGAA |
| GAPDH | GAGTCAACGGATTTGGTCGT | GACAAGCTTCCCGTTCTCAG |
