# Supplementary material for: EBV-Induced CXCL8 Upregulation Promotes Vasculogenic Mimicry in Gastric Carcinoma via NF-κB Signaling
Source: Front Cell Infect Microbiol. 2022 Mar 7;12:780416. doi: 10.3389/fcimb.2022.780416 (PMC8936189; doi:10.3389/fcimb.2022.780416)
Supplement: Supplementary file 2 [file DataSheet_2.docx]

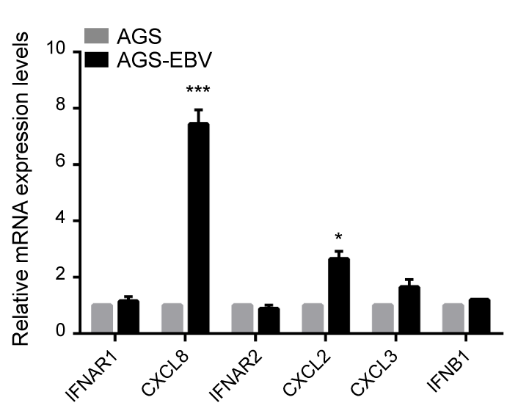


**Fig. S1.** qRT-PCR analysis of the cytokines in AGS and AGS-EBV cells carrying recombinant EBV, and CXCL8 displayed the greatest fold change in AGS-EBV group. Compared with AGS: *P<0.05, ***P<0.01.

*
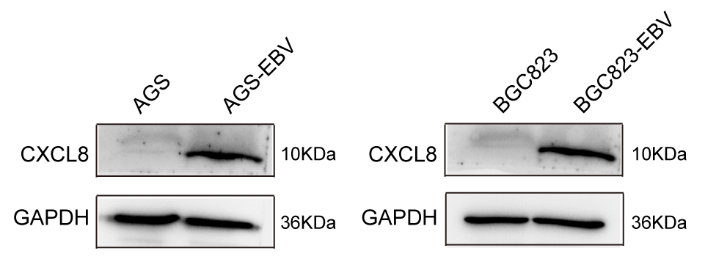
*

**Fig. S2.** **The protein level of expression of CXCL8 in gastric carcinoma cells.**

Western blotting analysis showed the expression of CXCL8 in gastric carcinoma cells.


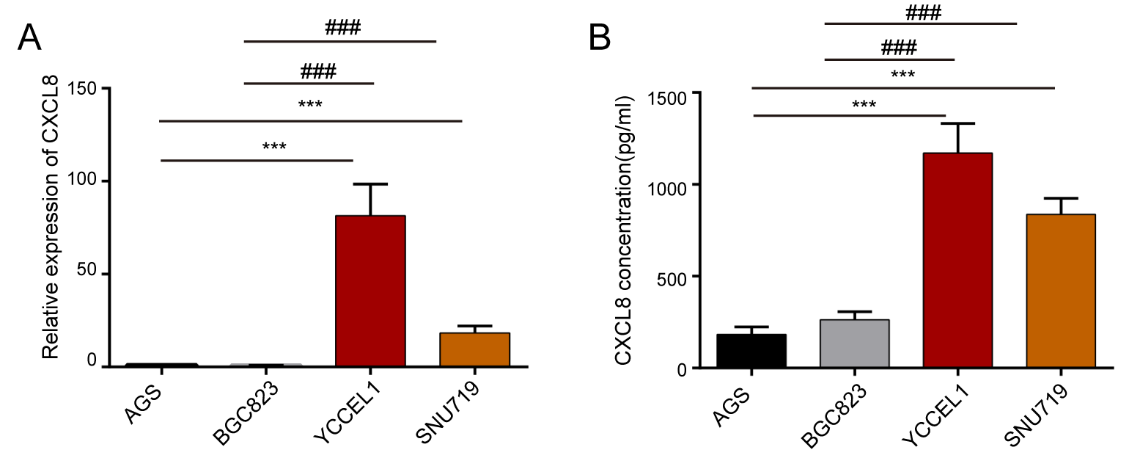


**Fig. S3. The level of expression of CXCL8 in naturally infected GC cell lines.**

(A) Relative mRNA expression of CXCL8 was assessed in paired EBV-negative and EBV-positive GC cells (AGS vs. and BGC823 vs. YCCEL1 and SNU719) by qRT‑PCR. CXCL8 mRNA level was higher in EBV positive cells than that in EBV negative cells. Compared with AGS: *P < 0.05, **P < 0.01, ***P < 0.001; Compared with BGC823: #P < 0.05, ##P < 0.01, ###P < 0.001.

(B) CXCL8 concentration in the supernatant of EBV− and EBV+ GC cells was quantified by ELISA at indicated times. CXCL8 level was higher in EBV positive cells than that in EBV negative cells. Compared with AGS: *P < 0.05, **P < 0.01, ***P < 0.001; Compared with BGC823: #P < 0.05, ##P < 0.01, ###P < 0.001.


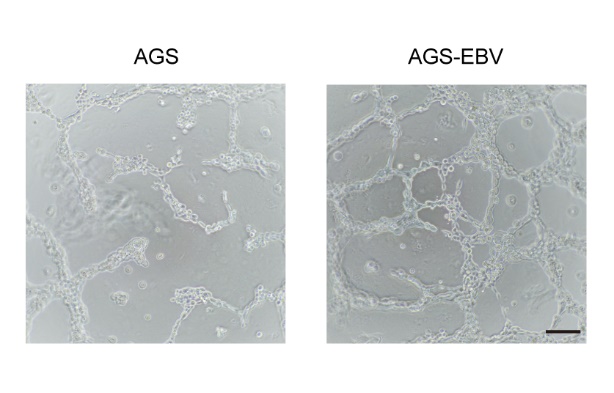


**Fig. S4.** Representative images of tube formation on Matrigel of AGS and AGS-EBV cells. Scale bars = 50 μm.

*
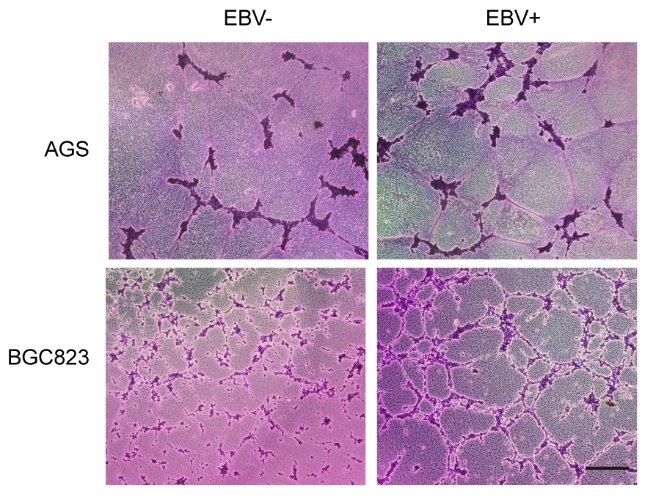
*

**Fig. S5.** PAS staining in EBV negative and EBV positive cells.

For PAS double staining, cells were seeded on coverslips, fixed with 4% paraformaldehyde, and incubated with the PAS for 20 min, and counterstained with hematoxylin. Scale bars = 100 μm.


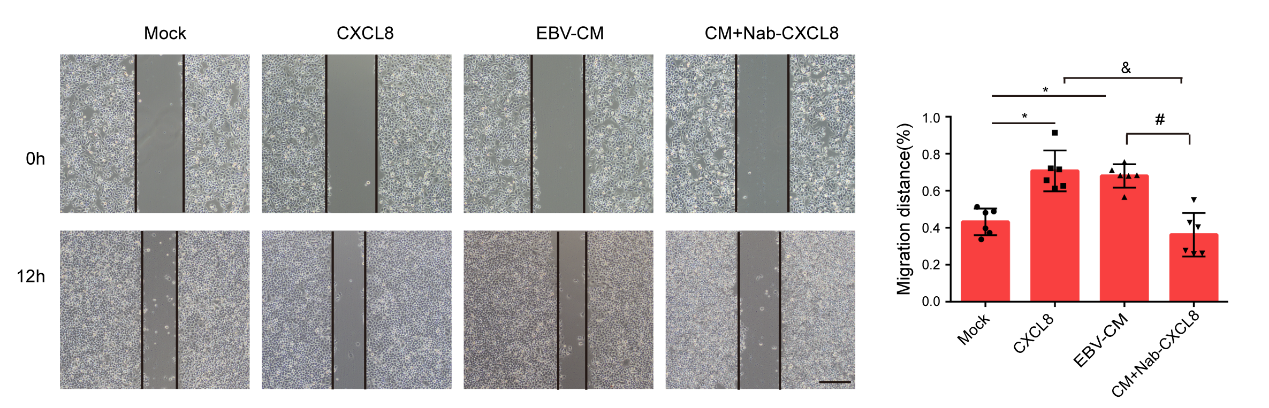


**Fig. S6.** Migration of BGC823 cells with different treatments as indicated were measured by wound healing assays. CXCL8:2ng/ml; EBV-CM: EBV conditioned medium; Nab-CXCL8: anti-human CXCL8 neutralization antibody (Nab), 1μg/mL for neutralization. Blocking CXCL8 by neutralization antibody (Nab) decreases the migration in GC cells. Scale bars = 100 μm. Compared with NC: *P<0.05; compared with CXCL8 or EBV-CM: ^#^P<0.01; compared with BAY11-7082: ^&^P<0.01.

*
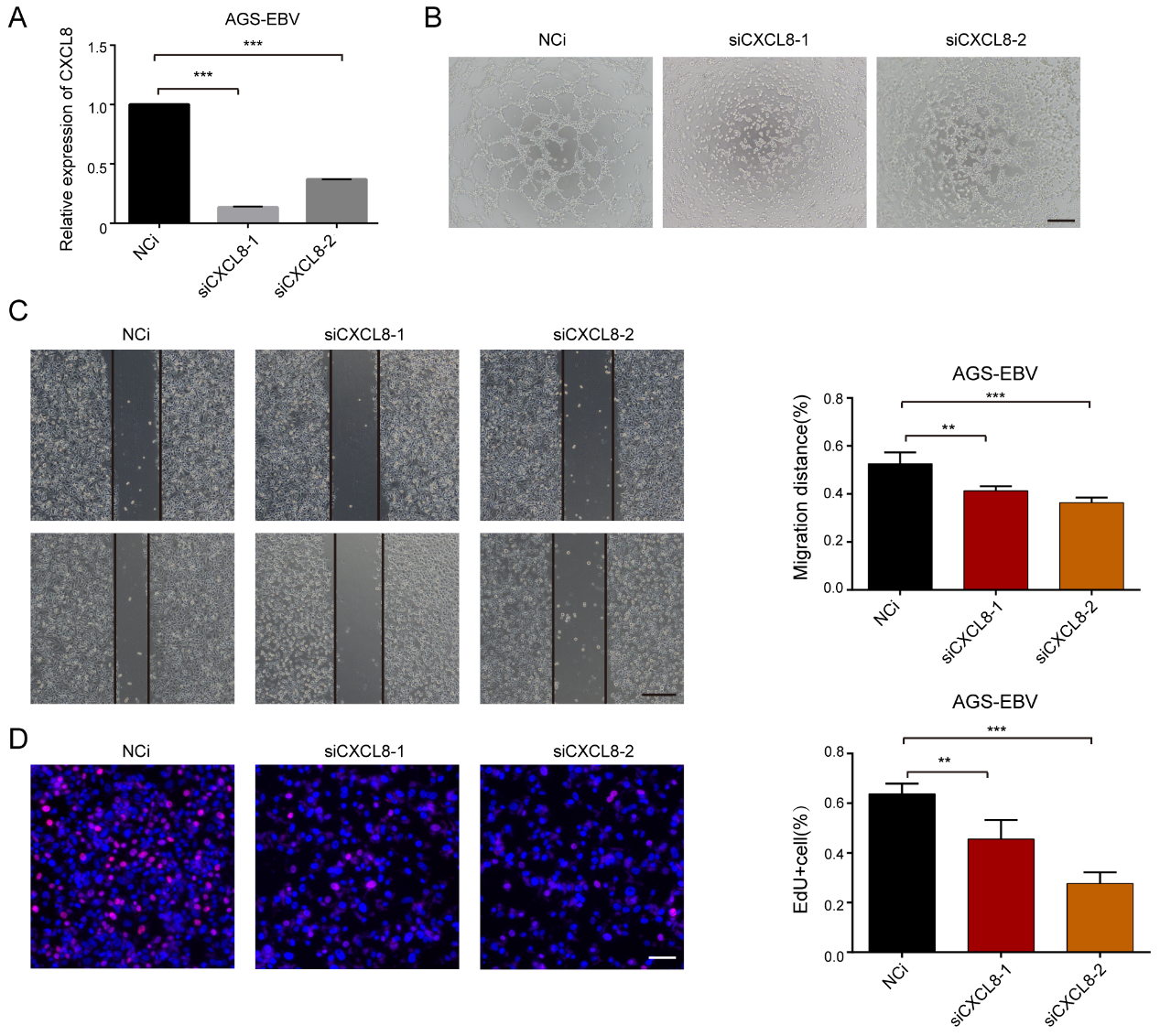
*

**Fig. S7.** CXCL8 is involved in VM formation.

(A) AGS-EBV cells were transfected with control or CXCL8-specific siRNAs. The knockdown efficiency was determined by qRT-PCR. Among the three siRNAs, si-1 and si-2 dramatically downregulated CXCL8 expression.

(B) AGS-EBV cells were transfected with NC or siRNA CXCL8. Representative images of tube formation on Matrigel of control and CXCL8 knockdown EBV positive cells. Tubes were counted with 100× magnification by Image J. Scale bars = 50 μm.

(C) Migration of AGC-EBV cells transfected with control or CXCL8-specific siRNA was measured by wound healing assays. Scale bars=100 µm.

(D) EdU assay assessed the proliferation of AGS-EBV cells transfected with control or CXCL8-specific siRNA. Scale bars = 50 μm. Compared with NCi: *P<0.05, **P<0.01, ***P<0.001.

*
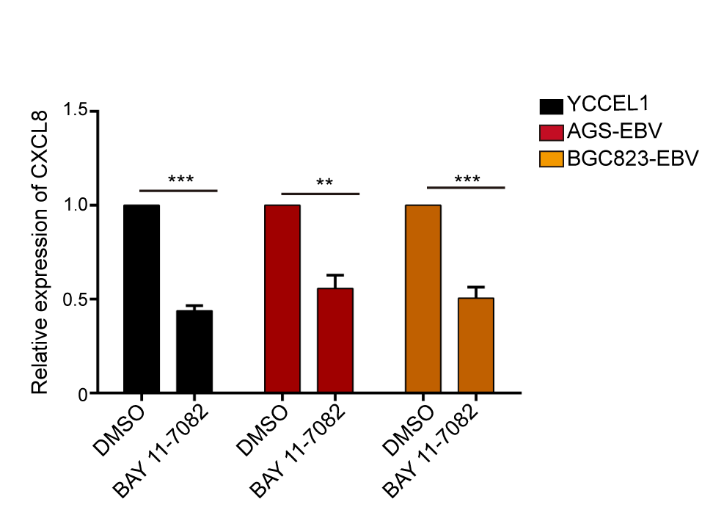
*

**Fig. S8.** The effect of the NF-kB inhibitor on CXCL8 production in naturally infected gastric carcinoma cell lines.

Relative mRNA expression of CXCL8 was assessed in EBV-positive GC cells (YCCEL1, AGS-EBV and BGC823-EBV) with the treatment of BAY 11-7082 by qRT‑PCR. *P < 0.05, **P < 0.01, ***P < 0.001.


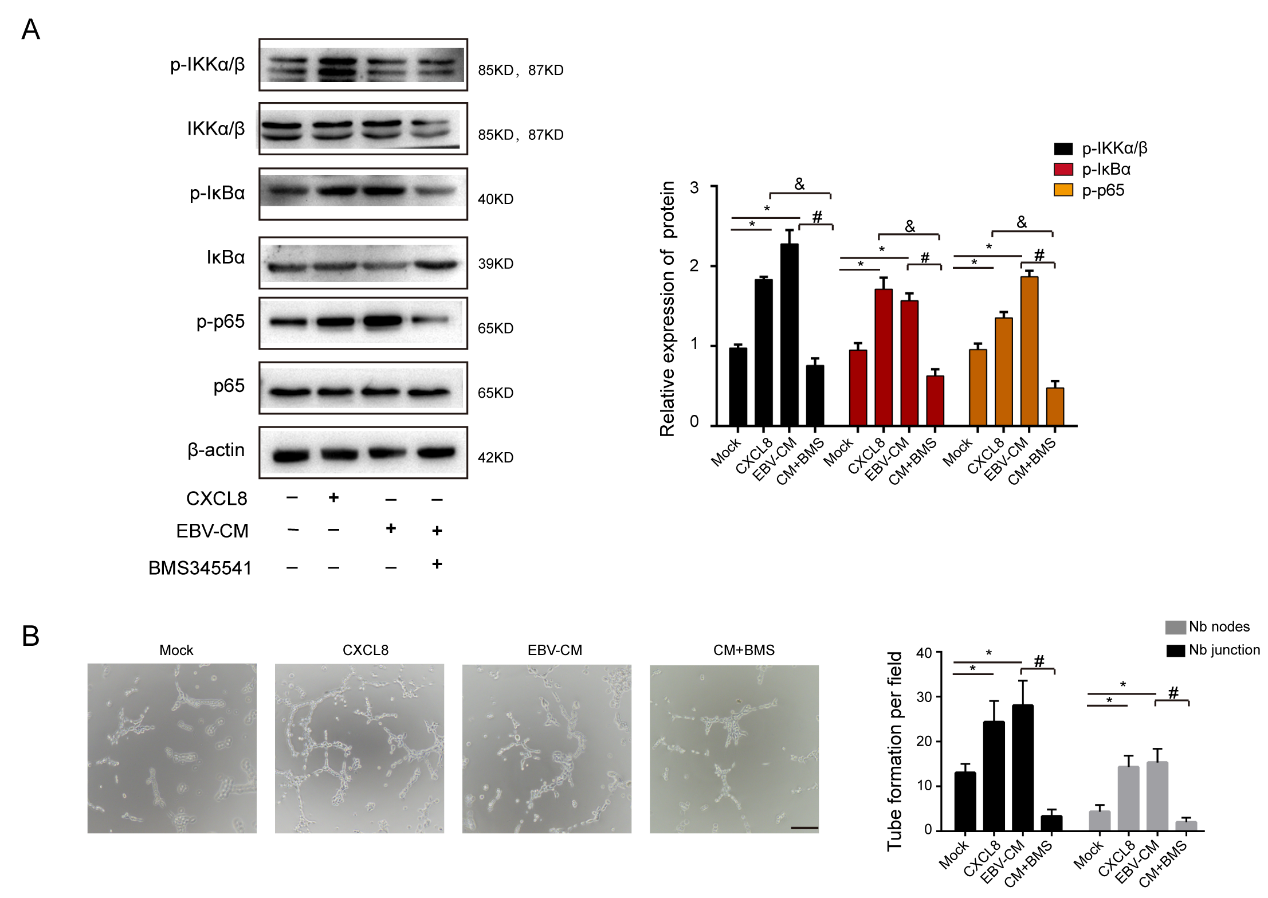


**Fig. S9.** NF-κB signaling participated in VM formation induced by CXCL8.

(A)The expressions of NF-κB signaling molecules in GC cells with different treatments as indicated were determined by immunoblotting. The expression of p‑IκBα and p‑NF-κB in AGS cells was upregulated with the treatment of recombinant human CXCL8 as well as EBV-CM, which was reversed by BMS (5 µM). BMS: BMS345541, an NF-κB inhibitor. β-Actin was used as a loading control. Compared with NC: *P<0.05; compared with +CXCL8 and +EBV-CM: #P<0.01; compared with BMS345541: &P<0.01.

(B) Representative images (left) and quantification (right) of tube formation on Matrigel with different treatments as indicated. BMS345541 (5 µM) decreased VM formation. Tube numbers were counted with 100× magnification by Image J. Scale bars = 50 μm. Compared with NC: *P<0.05; compared with +CXCL8 and +EBV-CM: #P<0.01; compared with BMS345541: &P<0.01.


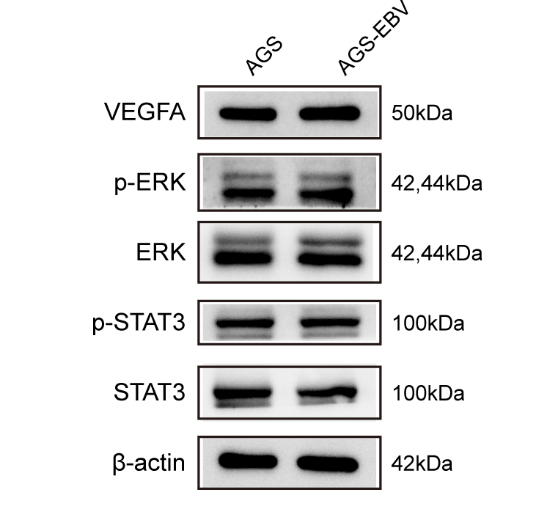


**Fig. S10.** AGS and AGS-EBV cells were lysed in SDS-loading buffer, and VEGFA, ERK level and STAT signaling activity were determined by immunoblotting.


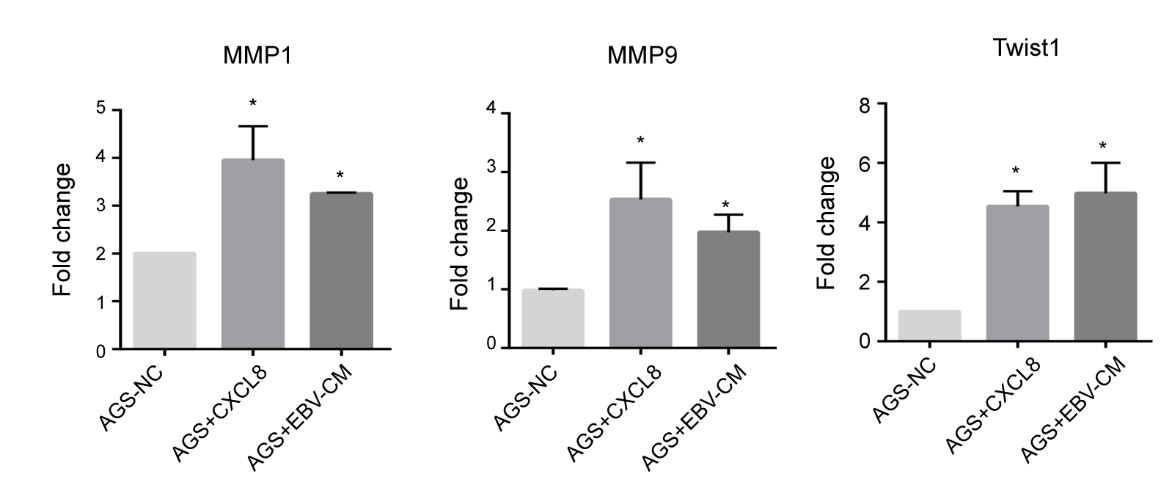


**Fig. S11.** Expression of VM-promoting related genes, MMP1, MMP9 and Twist1, were assessed by qRT‑PCR. Compared with NC: *P<0.05.

*
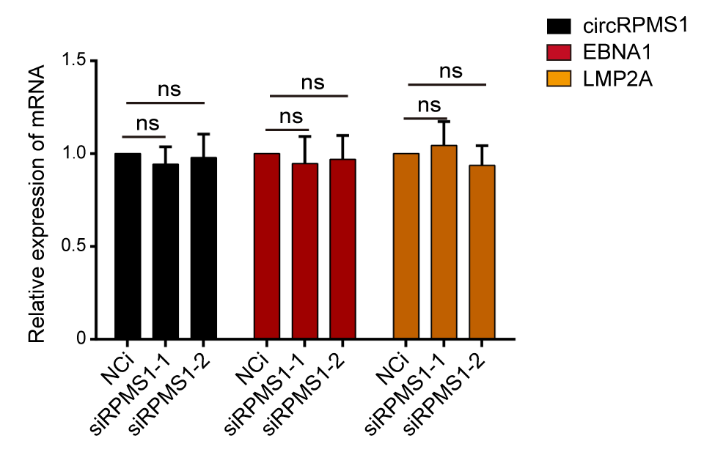
*

**Fig. S12.** The effect of the RPMS1 siRNA on the expression of other EBV latent genes.

Relative mRNA expression of EBV latent genes (circRPMS1, EBNA1, and LMP2A) was assessed by qRT‑PCR in control (NCi) and RPMS1 knockdown (siRPMS1) EBV positive cells.


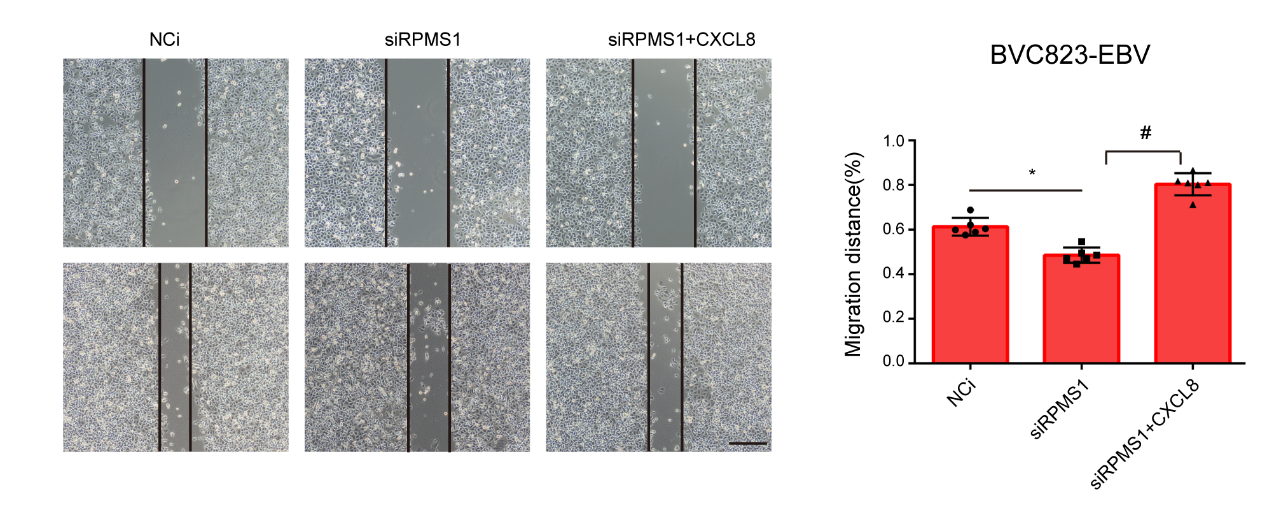


**Fig. S13.** Migration of BGC823-EBV cells transfected with control or RPMS1-specific siRNA was measured by wound healing assays. Scale bars=100 µm. Compared with NC: *P<0.05, **P<0.01; compared with siRPMS1: ^#^P<0.01.


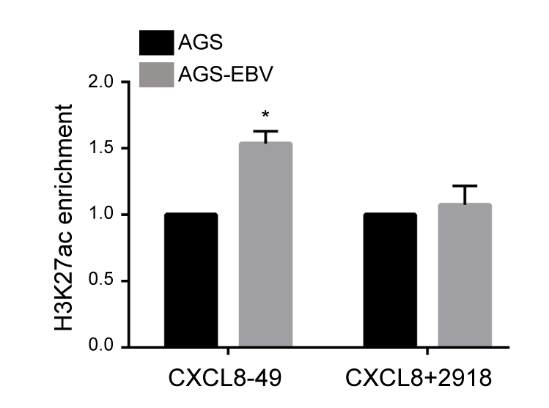


**Fig. S14.** ChIP analysis of CXCL8 promoter and downstream DNA with H3K27ac antibodies in AGS and AGS-EBV cells. *P<0.05.


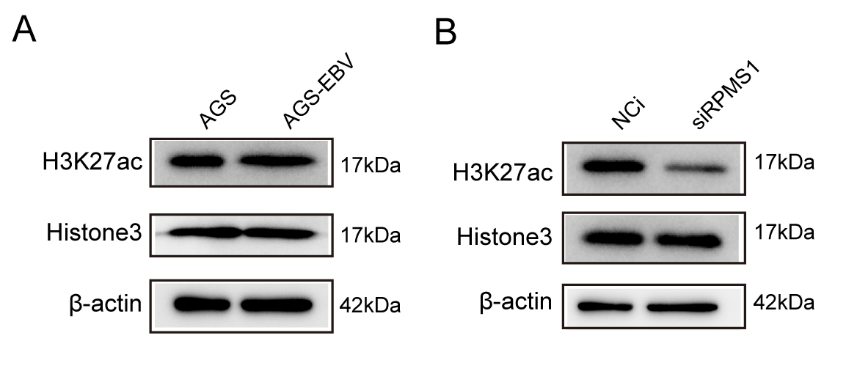


**Fig. S15.** (A) AGS and AGS-EBV cells were lysed in SDS-loading buffer, and the H3K27ac level was determined by immunoblotting.

(B) AGS-EBV cells were transfected with control or siRPMS1-specific siRNAs. The H3K27ac level was determined by immunoblotting.
